# Biyuanling suppresses the toluene-2, 4-diisocyanate induced allergic rhinitis in guinea pigs

**DOI:** 10.18632/oncotarget.23039

**Published:** 2017-12-08

**Authors:** Meixian Xiang, Li Wu, Hanwen Su, Bing Han, Huanxiang Liu, Xincai Xiao, Xian Yin, Ya Fan, Lang Zhang, Yuying Huang, Lei Zhao, Guangzhong Yang

**Affiliations:** ^1^ School of Pharmaceutical Sciences, South-Central University for Nationalities, 430074, Wuhan, PR China; ^2^ Renmin Hospital of Wuhan University, 430060, Wuhan, PR China; ^3^ Department of Pathology, Pennsylvania State University College of Medicine, Hershey, PA, 17033, USA; ^4^ Department of Infectious Diseases, Union Hospital, Tongji Medical College, Huazhong university of Science and Technology, Wuhan, China, 430022, PR China

**Keywords:** BLG, allergic rhinitis, SP, TNF-α, VCAM-1

## Abstract

Allergic rhinitis (AR), one of the common diseases of the upper respiratory system, is associated with high risk of nasopharyngeal carcinoma. Biyuanling Granules (BLG), a formulated preparation of traditional Chinese medicine, has been used in China for treatment of AR for more than a decade; however, its exact action against allergic rhinitis and the mechanism involved remain unclear. In this study, we studied the effects of BLG on allergic rhinitis induced by toluene-2, 4- diisocyanate (TDI) in guinea pigs. The anti-AR effects of BLG were evaluated by behavior observations, histological examinations of the nasal tissues stained with hematoxylin and eosin staining (H&E), immunohistochemical analyses of pulmonary surfactant associated protein (SP), Bcl-2 Associated X Protei (Bax), tumor necrosis factor (TNF-α) and vascular cell adhesion molecule-1 (VCAM-1) in the nasal mucosa, and serum tests of interleukin-4 (IL-4) and human SARS-specific immunoglobulin (SIgE) levels. We observed that in the AR-positive animals treated with BLG, the symptom scores were significantly higher (*P* < 0.01), the nasal mucosa edemas and inflammatory infiltrates were significantly alleviated (*P* < 0.01) and the serum IL-4 and SIgE levels were significantly decreased (*P* < 0.05) as compared with the control group. Immunohistochemical examinations of the nasal mucosa demonstrated that the expressions of TNF-α, SP and VCAM-1 were significantly decreased (*P* < 0.01), whereas Bax expression was increased in the BLG treatment groups (*P* < 0.05). These results indicate that BLG can effectively suppress the TDI-induced AR, and that the protective effects of BLG were associated with reductions of TNF-α, SP and VCAM-1, and an elevation of Bax, suggesting that BLG exerts its AR-suppressive effects through inhibition of inflammatory response.

## INTRODUCTION

Rhinitis is a common disease occurring in the upper respiratory system and has no significant seasonal association [1]. In China, there are approximately 37% of the total population that suffers from rhinitis in their life span. This disease has no age or gender prevalence, and can occur in children, adults and elders [2]. As environmental pollutions have become a serious problem in China and some other developing counties, an increasing morbidity is thus predictable [3]. Clinically, rhinitis can be classified as acute rhinitis, chronic rhinitis, medication-induced rhinitis, allergic rhinitis (AR) [4–7] and atrophic rhinitis [8]. Notably, it has been reported that AR can be casually associated with subsequent nasopharyngeal carcinoma (NPC), and that patients with repeated medical visits for AR had a higher risk for NPC if they were not treated effectively [9].

The management of rhinitis generally consists of systemic and local treatments [10]. The systemic treatments include identification and avoidance of allergic materials, applications of antibiotic agents, anti-fungal, anti-inflammatory and anti-allergic medications, as well as managements of systemic chronic diseases [11]. The local treatments include nasal cavity draining and cleaning, local application of steroid preparations and decongestive reagents, pain alleviation and surgical treatments [12]. Dozens of medical preparations including several traditional Chinese medicines have been used for relief of the symptoms of AR, and among them, Biyuanling Granules (BLG), a formulated preparation of traditional Chinese medicine consisting of Xanthium sibiricumPatrinexWidder (Xanthiumae) [13], Lonicera japonica (Lonicerae) [3], Glycyrrhiza uralensis Fisch (Glycyrrhizae) [14] and Scutellariabaicalensis Georgi (Scutellariae) [15], has been shown to be effective for acute, chronic, atrophic and AR, and to have mild side effects. We previously reported that BLG has significant anti-inflammatory and analgesic effects, and can reduce the damage of acute lung injury [16]. However, the mechanisms underlying the anti-AR action of BLG remain unclear. Here, using a guinea pig model in which AR was induced by TDI (Toluene-2, 4- diisocyanate), we demonstrated that BLG effectively suppressed the TDI-induced AR, and the protective effects of BLG were associated with reductions of TNF-α, SP and VCAM-1, and an elevation of Bax. Dexamethasone (DEX) [17, 18] is widely used clinically in the treatment of patients with AR. Therefore, we used this agent as a positive control drug in this study. Our investigation underscores the potential of BLG as an effective medicinal preparation for treating patients with AR.

## RESULTS

### BLG treatment improved symptom scores in guinea pigs with AR

Table 1 shows that as compared with that of the normal control group, the symptom scores in all of the AR groups were increased by approximately 5 points (*P* < 0.01) before BLG treatment, indicating successful establishments of AR models. Following treatment with various doses of BLG, the symptom scores in all the treated groups were significantly decreased (*P* < 0.01 and *P* < 0.05), as compared with the AR group that received vehicle. DEX was used as a positive control. These observations indicate that BLG treatment can improve the behavioral performance of guinea pig suffering from AR.

**Table 1:** Animal behavior scores before and after treatment

Groups	Total	
	Pre-administration	administration
Control	2 ± 0.6	2 ± 0.5
AR	6.8 ± 0.5^##^	7.1 ± 0.9
BLG (16.0 mg/g)	6.6 ± 0.7^##^	4.1 ± 0.4^**^
BLG (8.0 mg/g)	6.5 ± 0.8^##^	4.7 ± 0.9^**^
BLG (4.0 mg/g)	6.5 ± 1.0^##^	5.2 ± 0.5
DEX	6.4 ± 0.8^##^	3.8 ± 0.7^**^

### Inhibitory effects of BLG on nasal tissue edema and inflammatory infiltrates of nasal mucosa

**Figure 1:**
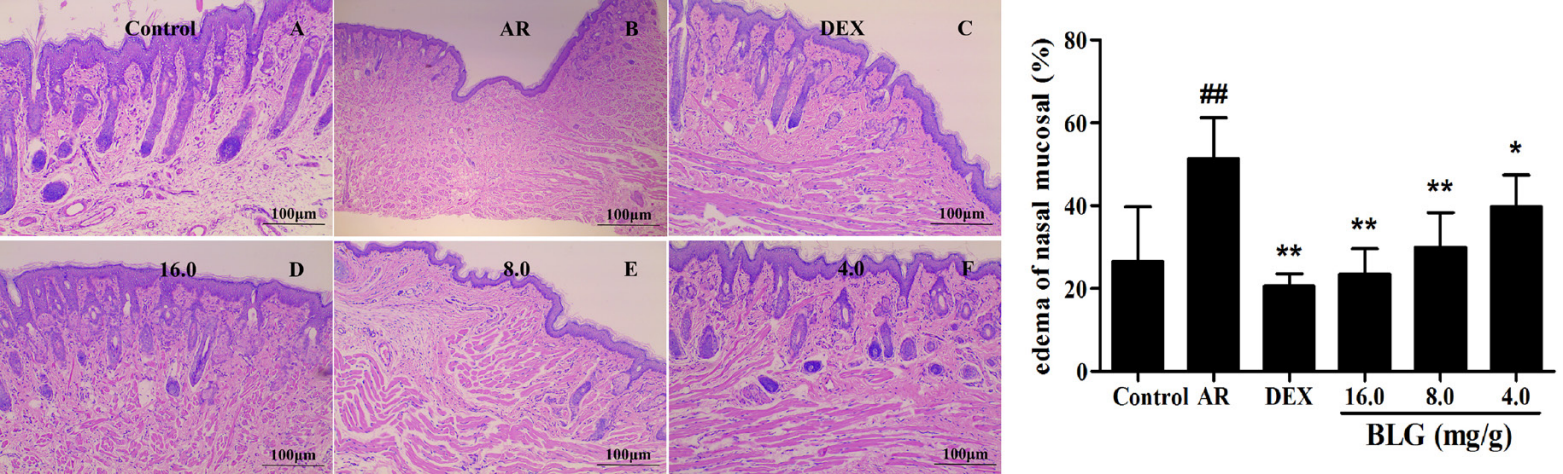
Effects of BLG on nasal tissue edema The edema areas in AR group were significantly increased when compared with those in normal control group (*P* < 0.01). Compared with the AR group, the edema areas in BLG and DEX treatment groups were significantly decreased (*P* < 0.01 and *P* < 0.05), indicating that the BLG could relieve the nasal tissue edema. Values were expressed as Mean ± SD (*n* = 10). ^*^P < 0.05 and ^**^P < 0.01 versus the AR group; ^##^*P* < 0.01 versus the control group.

To evaluate the effects of BLG on nasal edema, the Mias-2000 image analysis system was used to analyze the edema site area accounts to nasal lamina propria ratio. The edema areas of the AR group were significantly increased as compared with the normal control group (Figure 1). By contrast, all of the AR animals treated with BLG showed a significant decrease in edema areas (Figure 1). DEX was used as a positive control. These results indicate that BLG has strong inhibitory effects on nasal tissue edema.

**Figure 2:**
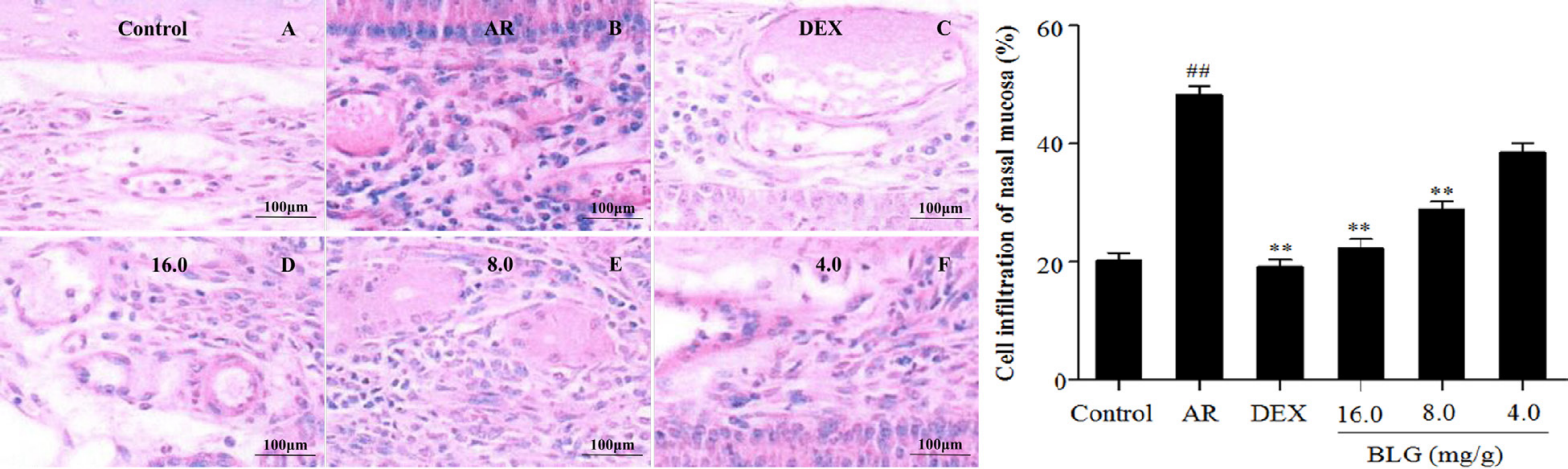
Effects of BLG on cell infiltration of nasal mucosa The inflammatory infiltration areas of the AR group were significantly higher than those of normal control group (*P* < 0.01). The Inflammatory infiltration areas significantly decreased in BLG and DEX treatment groups (*P* < 0.01) with exception of the Low dose group (4.0 mg/g) in which the decrease of inflammatory infiltration areas were not statistically significant. Values were expressed as Mean ± SD (*n* = 10). ^*^P < 0.05 and ^**^P < 0.01 versus the AR group; ^##^*P* < 0.01 versus the control group.

Next, we evaluated the inflammatory infiltrates of the nasal mucosa using the M2000 assay. Figure 2 showes that the inflammatory infiltration areas of the AR group without BLG treatment were significantly higher than those of the normal control group (*P* < 0.01). Treatment of the AR animals with 16 mg/g or 8.0 mg/g, or with DEX (positive control) significantly reduced the inflammatory infiltration areas (*P* < 0.01). Low dose (4.0 mg/g) of BLG caused an insignificant decrease of the inflammatory infiltration areas.

### Inhibitory effects of BLG on the levels of IL-4 and SIgE

**Figure 3:**
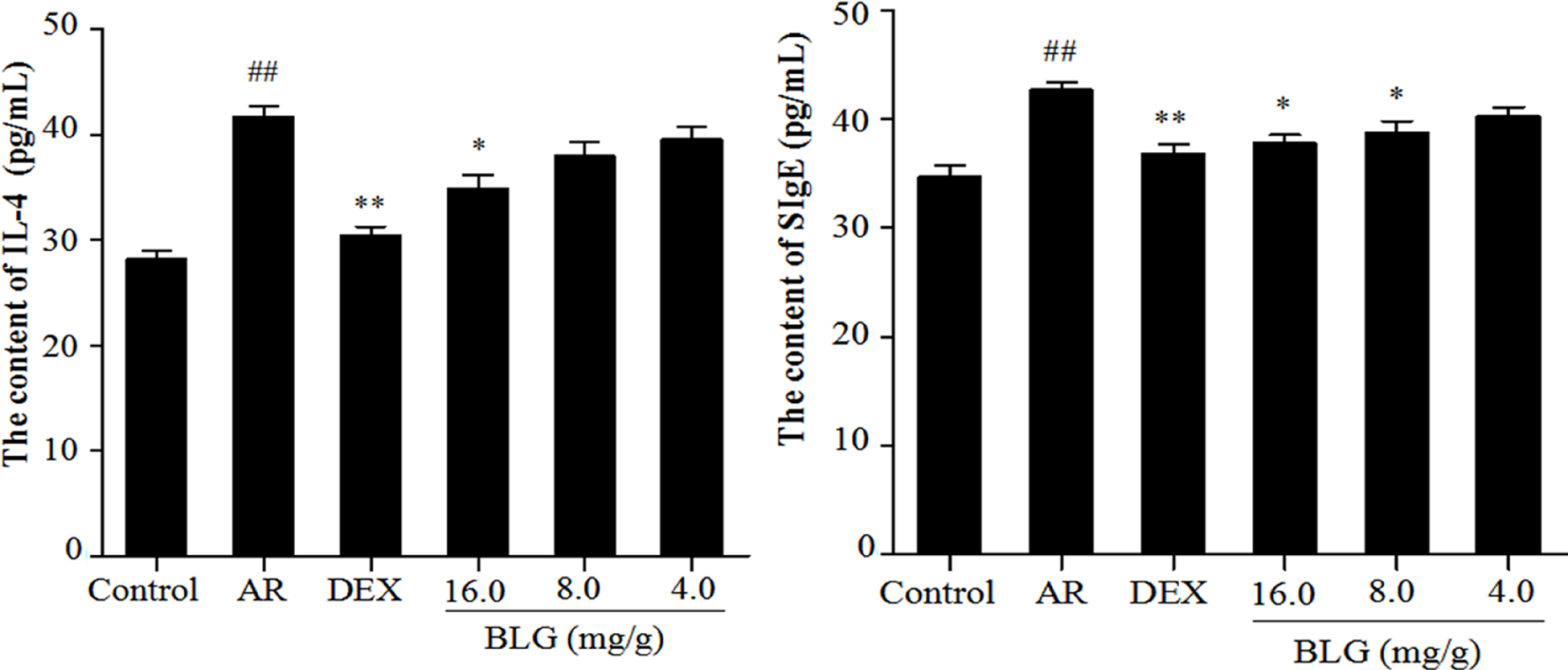
A Effects of BLG on the content of IL-4 and SIgE in guinea pig serum The levels of IL-4 in the serum in the AR group animals were significantly increased than those in normal control group animal (*P* < 0.01). The IL-4 levels in the treatment group animals were significantly decreased in the high dose group (16.0 mg/g) and DEX group (*P* < 0.05 and *P* < 0.01) as compared with AR group. Values were expressed as Mean ± SD (*n* = 10). ^*^P < 0.05 and ^**^P < 0.01 versus the AR group; ^##^*P* < 0.01 versus the control group. Figure 3B Effects of BLG on the content of SIgE in guinea pig serum. The levels of SIgE in the serum of the AR group animals were significantly higher than those in the normal control group (*P* < 0.01). The SIgE levels intreatment group animals were significantly decreased in high does group (16.0 mg/g) and mid group (8.0 mg/g) and DEX group as compared with the AR group (*P* < 0.05 and *P* < 0.01). The decrease in SIgE levels was not statistically significant in low dose group. Values were expressed as Mean ± SD (*n* = 10). ^*^P < 0.05 and ^**^P < 0.01 versus the AR group; ^##^*P* < 0.01 versus the control group.

The animals with AR had a significantly higher level of IL-4 in the serum than those normal animals (Figure 3A). BLG at 4.0 or 8.0 mg/g barely had any effects on IL-4 level (Figure 3A). However, treatment of AR animals with 16.0 mg/g of BLG caused a significant decrease of IL-4 levels (Figure 3A). Similarly, the levels of SIgEin the AR animals were significantly decreased following treatment with 16.0 mg/g or 8.0 mg/g of BLG (Figure 3B). DEX was used as a positive control.

### Effects of BLG on the expression of TNF-α, SP, Bax and VCAM-1 and P65

**Figure 4:**
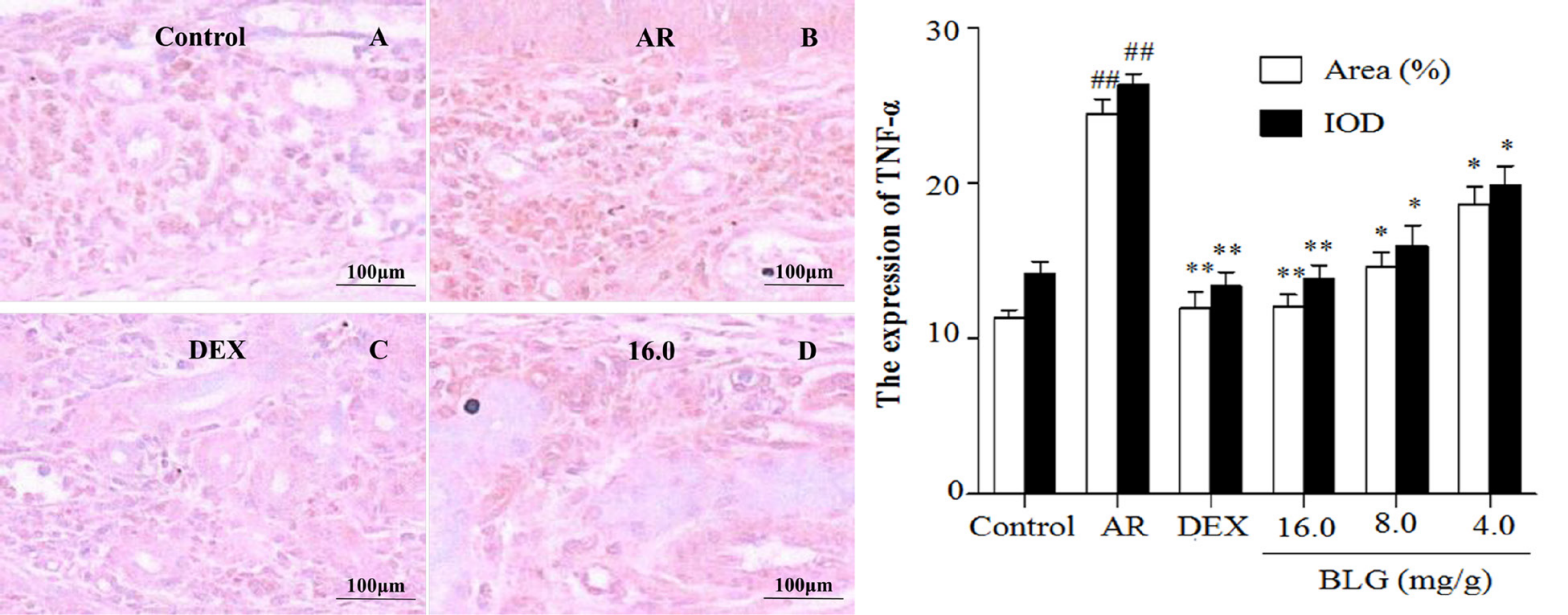
Effects of BLG on the expression of TNF-α in guinea pig nasal mucosa The IOD and the area ratio of TNF-α was significantly increased in the AR group when compared with the normal control group (*P* < 0.01). The BLG and DEX treatment groups showed significant decrease when compared with the AR group (*P* < 0.01 and *P* < 0.05). Values were expressed as Mean ± SD (*n* = 10). ^*^P < 0.05 and ^**^P < 0.01 versus the AR group; ^##^*P* < 0.01 versus the control group.

**Figure 5:**
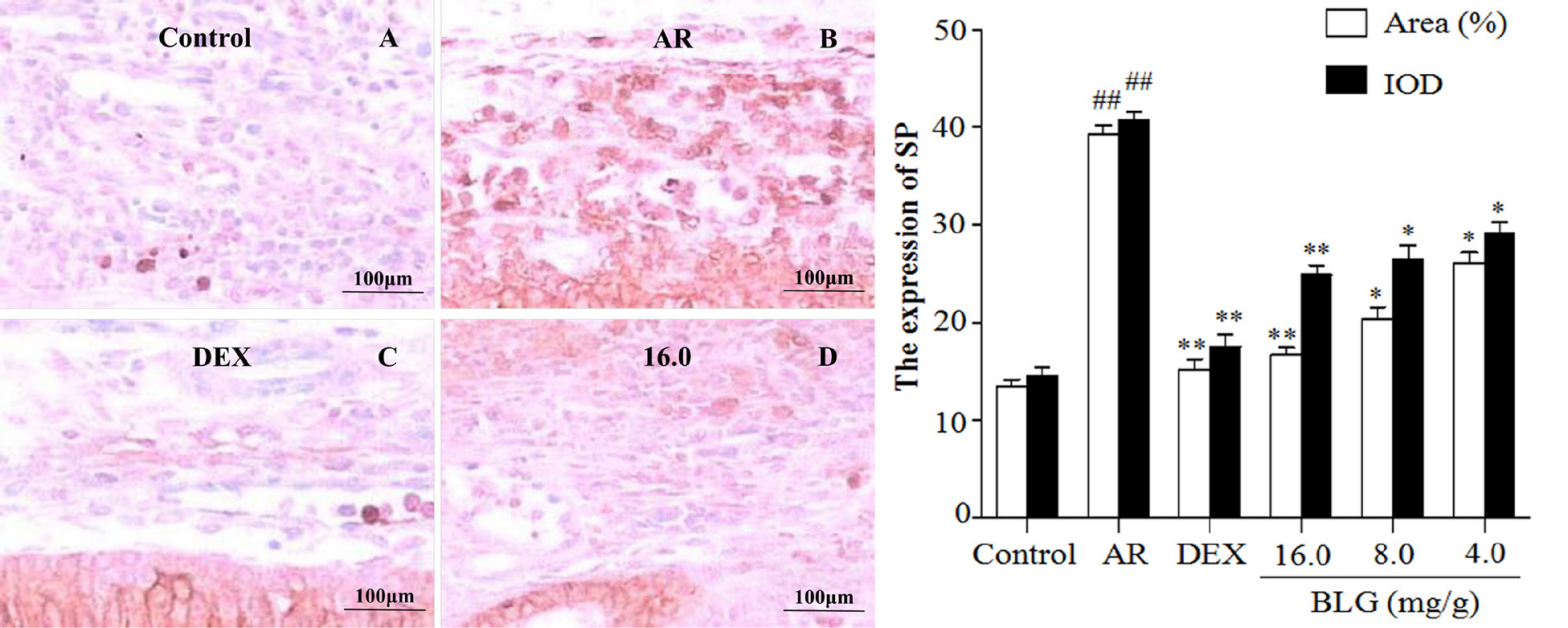
Effects of BLG on the expression of SP in guinea pig nasal mucosa The IOD and the area ratio of SP of the AR group was significantly higher than they were in the control group (*P* < 0.01). The BLG and DEX treatment groups showed significant decrease when compared with AR group (*P* < 0.01 and *P* < 0.05).Values were expressed as Mean ± SD (*n* = 10). ^*^P < 0.05 and ^**^P < 0.01 versus the AR group; ^##^*P* < 0.01 versus the control group.

**Figure 6:**
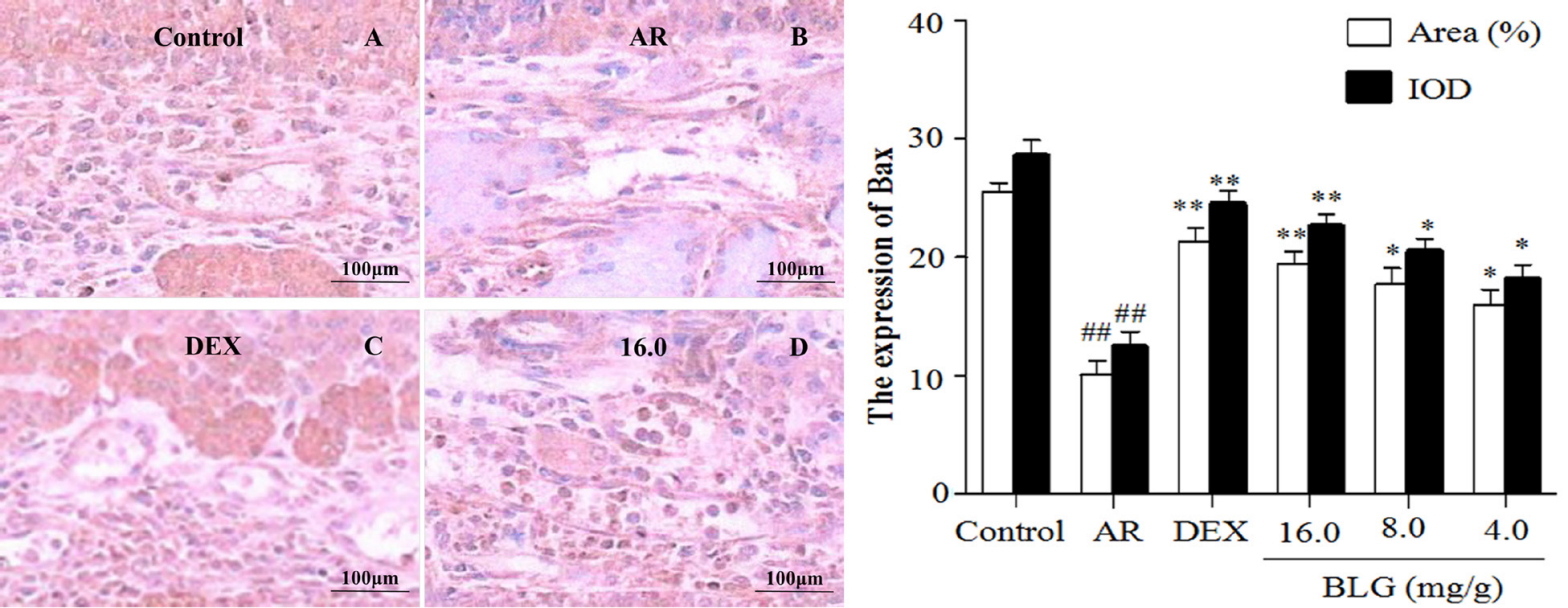
Effects of BLG on the expression of Bax in guinea pig nasal mucosa The IOD and the area ratio of Bax was significantly decreased in the AR group when compared with the normal control group (*P* < 0.01). The BLG and DEX treatment groups had a significantly increased expression when compared with AR group (*P* < 0.01 and *P* < 0.05). Values were expressed as Mean ± SD (*n* = 10). ^*^P < 0.05 and ^**^P < 0.01 versus the AR group; ^##^*P* < 0.01 versus the control group.

**Figure 7:**
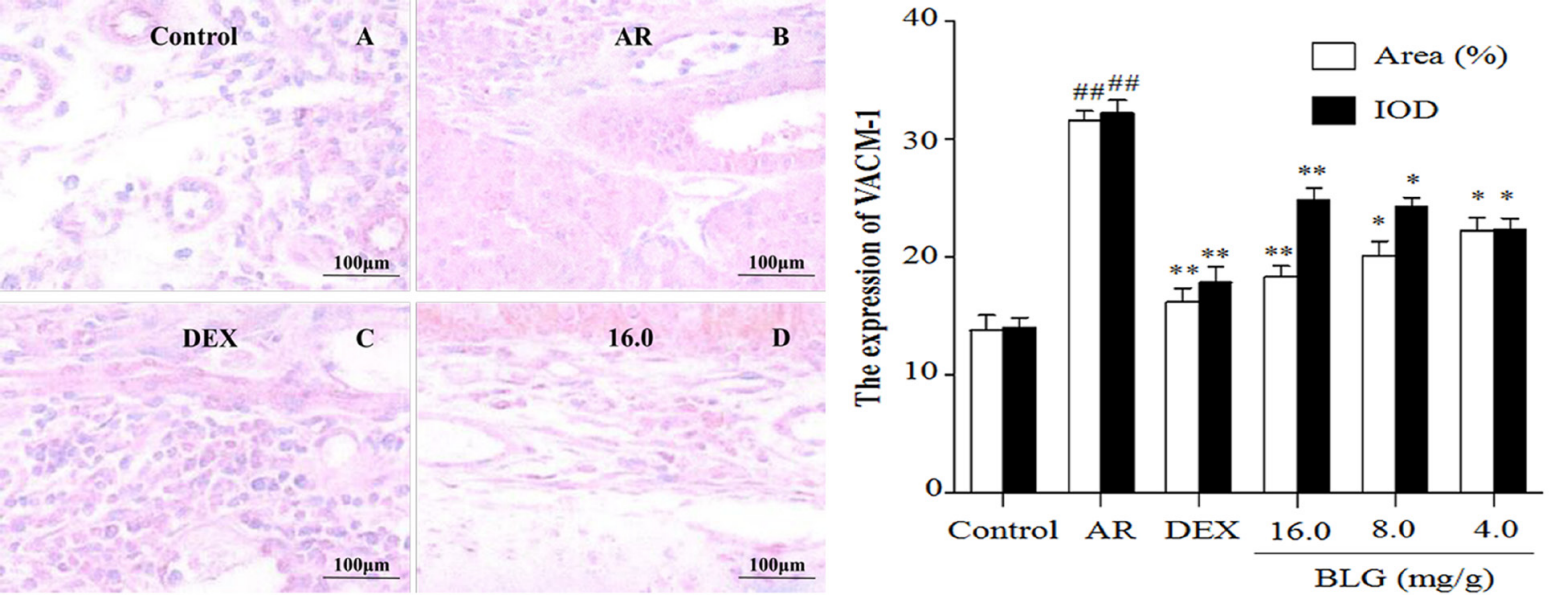
Effects of BLG on the expression of VCAM-1in guinea pig nasal mucosa The IOD and the area ratio of VCAM-1 was significantly increased in the AR group when compared with the normal control group (*P* < 0.01) and significantly decreased when compared with the AR group. The BLG and DEX treatment groups had a significantly increased expression when compared with AR group (*P* < 0.01 and *P* < 0.05). Values were expressed as Mean ± SD (*n* = 10). ^*^P < 0.05 and ^**^P < 0.01 versus the AR group; ^##^*P* < 0.01 versus the control group.

**Figure 8:**
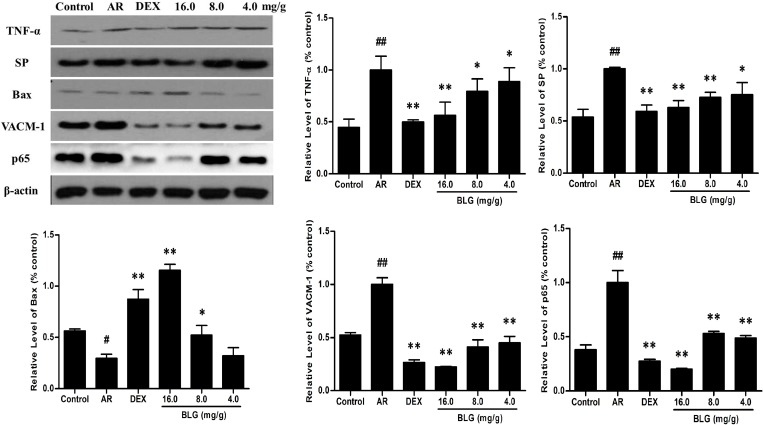
The protein levels of TNF-α, SP, Bax, VACM-1 and P56 in guinea pig nasal mucosa detected by western blot The expressions of TNF-α, SP and VACM-1 in nasal mucosae were significantly increased (*P* < 0.01) and that of Bax significantly decreased in the AR group (*P* < 0.01) compared with the normal control group. The expressions of the TNF-α, SP and VACM-1 and P56 in nasal mucosae in the BLG and DEX treatment groups were significantly decreased and that of Bax significantly increased when compared with the AR group (*P* < 0.01 and *P* < 0.05).Values were expressed as Mean ± SD (*n* = 10). ^*^P < 0.05 and ^**^P < 0.01 versus the AR group; ^##^*P* < 0.01 versus the control group.

The levels of TNF-α, as measured by the area ratio of the selected region in the nasal lamina propria, were significantly elevated in the AR animals as compared with the normal control animals (Figure 4 and Figure 8); notably, BLG or DEX treatment significantly decreased the TNF-α levels in the AR animals (Figure 4 and Figure 8). BAX protein was significantly decreased in the AR animals, but BLG or DEX treatment significantly increased the levels of Bax in the AR animals (Figure 5 and Figure 8). By contrast, the levels of SP were elevated in the animals with AR, as compared with the normal control animals (Figure 6 and Figure 8), but BLG or DEX treatment resulted in significant reductions in the expression of SP in the AR groups (Figure 6 and Figure 8). Similar effects of BLG on the expression of VCAM-1 in the AR animals were observed (Figure 7 and Figure 8).

## DISCUSSION

As an immunoglobin-mediated immunologic response, AR is a nasal mucous membrane-related disease caused by airborne allergens such as dust, pollens, or animal dander. Once the individuals who have sensitized immune system inhale allergens, it would produce degranulation of mast cells with releasing chemical mediators [19]. If the patients could not get prompt treatment, AR would worsen their life quality and lead to a significant higher healthcare costs. It is believed that those inflammatory processes can increase the risk of cancer if the patients are not treated effectively. Extensive investigations have been exploring the association between AR and NPC [20, 21]. However, the exact molecular mechanisms involved remain unclear. One possible explanation is that chronically repeated inflammation and stimulation in airway may contribute to a malignant change [3].

BLG is a traditional Chinese medicine preparation consisting of xanthium sibiricumpatrinexwidder (xanthiumae), lonicera japonica (lonicerae), glycyrrhiza uralensisfisch (Glycyrrhizae) and scutellariabaicalensisgeorgi (scutellariae). BLG has significantly anti-inflammatory and analgesic effects and its main active ingredients arebelieved to be baicalin and chlorogenic acid [22]. In the current study, we used the TDI-induced guinea pig AR model, which showed typical AR characteristiccs including rhinorrhea, sneezing and rhinobyon, and the pathological changes similar to that of patients, to demonstrate the inhibitory effect of BLG on AR [23].

The results of our study show that BLG effectively alleviated the symptoms of AR, and this was apparently associated with its effects on the immune system, as evidenced by decreases of the releasing of inflammatory mediators IL-4 and SIgE, inhabiting mast cells degranulation and intramucosal inflammatory cell infiltration, suppressing the expressions of inflammation-related proteins SP, TNF-α, VCAM-1, and elevating the levels of apoptosis-related protein Bax. The development of AR is a rather complicated process involving sensitization by allergens, expression of antigen specific IgE, degranulation of mast cells and release of inflammatory mediators, such as histamine, tryptase, prostaglandins and leukotrienes, and intramucosal inflammatory cell infiltrations. TDI is a highly active chemical agent that can induce the activation of the mast cells, leading to a hypersensitive reaction in the nasal cavity of guinea pigs [24]. When treated with BLG at different doses, AR symptoms of the experimental animals were obviously alleviated [25]. Our results indicate that BLG powders can reduce local allergic reaction and achieve the therapeutic efficacy by alleviating tissue edema and rhinobyon. However, in this study, the drugs did not cause a complete recovery of guinea pigs in terms of the symptoms, which we think might be attributable to insufficient treatment duration.

Our study also showed that BLG powders could reduce the level of IL-4, a cytokine secreted by Th2 immune cells, which is one of the key factor responsible for hypersensitivity. The levels of IL-4 were reversibly parallel with BLG doses and reached statistically significance in animals treated with the high dose (16.0 mg/g). This result suggest that the AR symptom-reducing effects of BLG involve the re-balance of the immune regulating cytokines. IgE is one of the serum immunoglobulins with specific affinity with mast cells and eosinophiles and participates in the type I allergic reaction as a mediator [25, 26]. For this reason, IgE can be used as one of the indicators for evaluating the therapeutic effects on AR. In their studies, Thakare V N etc [27] and Yu S etc [28] found that decrease in IgE levels can influence the mast cell degranulation so that to alleviate the symptoms of AR. Our study demonstrated that BLG powders reduced the levels of serum IgE in a dose dependent pattern, although the effects were observed in all doses groups. This result suggested that BLG can significantly inhibit the secreting/releasing of the inflammatory mediators, thereby producing the therapeutic effects. NF-κB signaling pathway can regulate the expression of inflammation-related proteins, in the inflammatory response process has played a key role [29]. Meanwh-ile, BLG can down-regulate the expression of inflammatory mediators by inhibiting NF-KB, and these molecular events are closely related to the mitigation effect of BLG on LPS-induced lung tissue response in ALI mice (the results were not showed) [30]. The expression of TNF-alpha is increased when the body undergoes an inflammatory process. Over expression of TNF-alpha inhibits the expression of the apoptosis protein bax, which subsequently leads to an accumulation of the inflammatory cells due to a delayed clearance. The membranes of the inflammatory cells also undergo a change in such situation, leading to an increase in permeability. The expression of the secretory mucinous cytokines VCAM-1 by the inflammatory cells is increased, causing the development of systemic interstitial inflammation from local inflammation. In meantime, the expression of neuropeptide SP is increased, further deteriorating the systemic inflammation through the neuroimmunomodulation loop. As one of the transcription factors, NF-KB plays an important role in inflammatory reactions by modulating the transcription and expression of inflammation related proteins TNF-alpha, Bax, VCAM-1, the neuropeptide SP, and by modulating the secretion levels of the immune related factors, such as IL-4 and SIgE.

TNF-α is involved in the synthesis of IgE by the lymphocytes and the conversion of IgE to IgG. It also induces the releases of VCAM-1 and inflammatory factors IL-1and IL-8 [31]. It was found that the expression of VCAM-1 was significantly increased in the nasal mucosae of the mice suffering from AR, suggesting that this protein is associated focal mucosal inflammatory infiltrates [32]. For this reason, expression of VCAM-1 can be used for studying the mechanism of anti-AR medications. In our study, the expressions of TNF-α and VCAM-1 were significantly decreased in the BLG treated animals, suggesting that its anti-AR actions were indeed associated with the reduction of the expressions of TNF-α and VCAM-1, which inhibit the secretions of IL-1 and IL-8.

SP is a neuromodulator involving in neuro-inflammatory reactions, which can bind to the receptors on the target inflammatory cells. SP can not only combine with the receptors on mast cells but also inhibit the proliferations of the lymphocytes, thus exacerbating AR through multiple pathways [33, 34]. Our results showed that the IOD and the area ratios of SP in all doses BLG treated groups were significantly lower than those in the AR control group. This result strongly argued that BLG could reduce the SP in the nasal mucosa of AR animals to alter the neuro-inflammation through multiple pathways.

Bax is one of the major members of Bcl-2 family, whose potential roles are to promote cell death (apoptosis) through permeabilization of mitochondrial outer membrane in response to different cellular stresses. It has been shown that Bax was expressed in the cytoplasm and nuclei of eosinophils and can promote apoptosis of the inflammatory cells to relief the symptoms of AR [35, 36]. We showed that the expressions of Bax in the BLG treatment groups are significantly higher when compared with the control group. These results suggested that Bax induced apoptosis of inflammatory cell may also play a role in anti- AR actions of BLG.

Transcription factor p65 also known as nuclear factor NF-kappa-B p65 subunit is a protein that in humans is encoded by the RELA gene [37] also known as p65, is a REL-associated protein involved in NF-κB heterodimer formation, nuclear translocation and activation. so, the protein levels of P65 was detected. The results showed that the expressions of P65 in the BLG treatment groups are significantly higher when compared with the control group.

Taken together, BLG can effectively alleviate the symptoms of TDL-induced AR, and its protective effects appear to be multifactorial and associated with the suppression on TNF-α, SP and VCAM-1, and the elevation of Bax. As BLG has no clinical side effects shown by some other anti-AR medications such as DEX, it may have better application prospect in treatment of AR and prevention of the AR-related diseases such as nasopharyngeal cancer.

## MATERIALS AND METHODS

### Reagents

BLG was supplied by the Department of Pharmacy, Renmin Hospital of Wuhan University (pharmaceutical batch number: Z20111009). TDI was purchased from Sigma. Co (USA, St. Louis, MO). Dexamethasone (DEX, Guangdong, China) (5 mg/kg body weight) was purchased from Sinopharm Chemical Reagent Co. Ltd., China. IL-4 Elisa kit and SIgE Elisa kit was obtained from Yuanye Bio-Technology (Shanghai, China). TNF-α (Tumor Necrosis Factor-α) antibody, SP (Substance P) antibody, Bax antibody and VACM-1 (Vasopressin-activated Ca(2+)-mobilizing-1) antibody were obtained from Bioss (Beijing, China). β-actin antibody were purchased from Beyotime (Shanghai, China). Goat anti-Rabbit IgG (Abcam, USA).

### Animals

Guinea pigs (180–220 g) were purchased from the Experimental Animal Center, Institute of Health and Epidemic Prevention (Wuhan, China). The animals were housed in a standard specific pathogen free (SPF) environment. All the animal experimental procedures were approved by the Animal Care and Use Committee of South-Central University for Nationalities (Wuhan, China) (Production license SKCK (E) 2015–0018, Hubei, China). All guinea pigs were housed under the same conditions in a temperature and humidity controlled room (20–25°C, 45%–75% relative humidity). Tap water and standard feed were provided on a regular basis.

### Establishment of a guinea pig model of allergic rhinitis

The guinea pigs (5-6 weeks, 180–220 g) were divided into control groups (*n* = 10) and experimental groups (*n* = 50). Sensitization and challenge protocol as described was applied with some modifications. The experimental groups of guinea pigs were dripped with 10% TDI olives solution from nostrils with micro pipette, 5μL each side, once daily for 7 days intranasal. In order to maintain a steady status in sensitized guinea pigs, it intranasal TDI after 15 min of administration every other day. The normal control group animals were treated with an equal volume of olive oil without TDI. BLG and dexamethasone was oral administration at the same time every day for a week.

50 guinea pigs were randomly selected form the successfully induced animals. They were divided into 5 groups with 10 guinea pigs in each of the following groups: model control group (positive control group, Labeled as AR in the Results), high dose group (16.0 mg/g), mid dose group (8.0 mg/g), low dose group (4.0 mg/g) and dexamethasone group (5 mg/kg). Ten of the guinea pigs treated with olive oil only were randomly selected as the normal control.

### Symptoms of AR and scoring

Symptoms of AR included sneezing, nose rubbing and rhinorrhea. Number of sneezing and nasal rubbing were counted continuously, and the rhinorrhea was calculated according to the amount of outflow. Sneezing was scored as 1-3 times for one point, 4-10 times for two points, more than 11times and above for three points; Nose rubbing was scored as continuous to nasal rubbing for 1 points and severe nasal scratching for 2 points. Rhinorrhea was scored as snot to the nostrils for one point, snot out of his nostrils for two points and face covered with snot for three points.

### Histopathologic evaluation

After the guinea pigs were sacrificed, the nasal mucosae were immediately harvested and fixed with 10% formaldehyde. Then the tissues were dehydrated, embedded in paraffin and sliced into 4μm sections. Hematoxylin and eosin staining was carried out with routine staining method. The pathologic changes of nasal mucosa were observed under the Mias-2000 image analysis system.

### Measurement of IL-4 and SIgE

Blood (5mL) was obtained by femoral artery phlebotomy and centrifuged for 20 min at 3000 rpm. Supernatant was removed and the sample was stored at −20°C. The concentrations of SIgE and IL-4 were measured using an ELISA Kit according to the manufacturer’s protocol.

### Measurement of SP, Bax, TNF-α, VCAM-1 in Nasal mucosa

The paraffin sections of nasal mucosa were deparaffinized and sections were treated with 3% hydrogen peroxide for 10 min. The sections were then blocked with 10% bovine serum albumin in PBS for another 10 min at room temperature. Next, the sections were incubated with primary antibodies overnight at 4°C. After thorough washing with PBS, the sections were incubated with the secondary antibodies for 1h at room temperature. Then the sections were stained with DAB after washed three times. The sections were then dehydrated washed and mounted. The levels of SP, Bax, TNF-α, VCAM-1 in nasal mucosa were evaluated under the Mias-2000 image analysis system.

### Western blot

Proteins were obtained from the nasal mucosal tissues of each guinea pig 6 h after the final intranasal provocation by using lysis buffer. The protein concentration was determined according to the BCA assay. The protein samples were subjected to SDS-PAGE, and transferred to PVDF membranes. After blocking with 5% bull serum albumin for 1 h, the membranes were incubated with primary antibodies overnight at 4°C (1:1000). The membranes were washed three times for 10 min each with washing buffer and incubated with the secondary antibodies for 1h at room temperature (1:10000). The target proteins were detected with ECL and grey value of bands were analyzed by image Lab software.

### Statistical analysis

The results were expressed as means ± SD and analyzed by one-way ANOSVA. Statistical significance was defined at *P* < 0.05.
